# LBP and iFABP mismatch in the evaluation of intestinal barrier dysfunction due to SARS-CoV-2 infection

**DOI:** 10.1016/j.clinsp.2025.100642

**Published:** 2025-04-23

**Authors:** Hermes Vieira Barbeiro, Denise Frediani Barbeiro, Heraldo Possolo de Souza, Francisco Garcia Soriano, Marcel Cerqueira Machado, Ludhmila Abrahão Hajjar

**Affiliations:** Laboratório de Investigação Médica da Disciplina de Emergências Clínicas, Departamento de Clínica Médica, Faculdade de Medicina da Universidade de São Paulo São Paulo, SP, Brazil

**Keywords:** COVID, Gut, Barrier, Inflammation, Elder

## Abstract

SARS-CoV-2 presents a hyperinflammatory scenario due to systemic inflammatory response syndrome with intense cytokine release, with consequent extrapulmonary involvement in 20 % of patients. The authors studied whether COVID-19 intestinal damage is a direct action of the virus on intestinal epithelial cells, with damage mainly at the tight junction. This is a retrospective observational study in a tertiary hospital emergency department. The authors studied 87 patients (46 patients over 61 years and 41 patients under 60 years old) with severe SARS-CoV-2 infection. The authors measured two plasma markers, LPS-Binding Protein (LBP) and ileal Fatty Acid-Binding Protein (iFABP). Furthermore, the authors evaluated the interaction between the two markers. TNF-α and IL-1 β were higher in bacterial co-infected patients and TNF-α was also higher in the older patients. Plasma iFABP levels were not statistically different in patients with bacterial co-infection; however, higher levels were found in the older population. Plasma LBP levels were higher in patients with bacterial co-infection when compared to patients without infection; however, when comparing plasma LBP levels in the older population with younger patients, no differences could be found. LBP, FABP, and cytokines can discriminate between bacterially infected patients and also discriminate elderly patients. The present study suggests that intestinal barrier dysfunction in SARS-CoV-2 infections is mainly due to damage to the intestinal tight junction complex with a disproportionately lower damage to enterocyte. In the older population, the authors also observed an increase in intestinal epithelial damage.

## Highlights

• Inflammation in SARS-CoV-2 has an extrapulmonary alteration in 20 % of cases.
• Intestinal damage is a direct action of the virus.
• Enterocyte damage is lower to tight junction alteration.

## Introduction

In December 2019, multiple cases of highly contagious viral pneumonia were reported in Wuhan Province of Hubei in China.^1^^,^^2^ A novel Coronavirus (SARS-CoV-2) was isolated and identified in the respiratory tract of infected patients.^2^ The World Health Organization named this disease as COVID-19, which spread rapidly around the world. COVID-19 usually enters the human body through the respiratory tract and gradually causes systemic disease.^1^

Severe COVID-19 is defined by the presence of dyspnea and hypoxemia.^1^ These characteristics are a consequence of the cells of the pulmonary epithelium being affected by the SARS-CoV-2 virus, causing intense local inflammation.^2^ This inflammatory process can produce lung damage and consequent respiratory failure, reducing blood oxygenation, and patients requiring endotracheal intubation and mechanical ventilation. In addition, many patients develop systemic inflammation that produces endothelial damage,^3^^,^^4^ increased thrombotic phenomena^5^ multiorgan failure^6^ and circulatory shock.^7^ Furthermore, there is a direct correlation between plasma levels of inflammatory markers and disease severity,^8^ suggesting that immune activation is primarily responsible for systemic involvement in COVID-19.^9^

In approximately 20 % of patients with SARS-CoV-2, an extrapulmonary hyperinflammatory state develops due to cytokine release syndrome.^10^ Gastrointestinal symptoms occur in 11 %‒20 % of COVID patients, and the damage to the intestinal mucosa may be a consequence of systemic inflammation. The breakdown of the intestinal barrier may lead to bacterial translocation from the intestine into the blood and mesenteric lymph, leading to further systemic inflammatory processes and the risk of developing bacterial sepsis.^11^^,^^12^ In addition to cytokines, released viral particles can directly damage the gastrointestinal tract by binding to angiotensin-converting enzyme type 2, its cell surface receptor, resulting in intestinal dysfunction and diarrhea. In the intestines, SARS-CoV-2 damages the tight junction and facilitates bacterial translocation.^13^ The literature has reported that the elderly population has more morbid and lethal SARS-CoV-2 infection. The gastrointestinal tract in the elderly population may be more vulnerable to SARS-CoV-2 infections, with few reports related to intestinal damage in the elderly population.^14^ Several studies have reported that SARS-CoV-2 infection compromises tight junction and intestinal barrier function. However, it is not known whether tight junction damage is due to intestinal epithelial cell injury or is limited to the tight junction alone.^13^^,^^15^

The aim of this study was to find out whether gut-specific biomarkers were elevated in patients with severe SARS, indicating that the gut was also affected by systemic inflammation. To clarify whether the intestinal barrier dysfunction occurring in patients with SARS-CoV-2 is due to epithelial injury, the authors measured plasma iFABP that is directly bound to the intestinal epithelium; and LBP that indicates bacterial translocation. LPS-Binding Protein (LBP), which evaluates the translocation process, and ileal Fatty Acid-Binding Protein (iFABP), which evaluates damage to intestinal epithelial cells. Intestinal Fatty Acid Binding Protein (iFABP) is a 15 kDa protein located at the tips of intestinal mucosal villi. This protein can appear in the blood after intestinal damage and, therefore, be used as a marker of intestinal damage.^16^^,^^17^ In addition, the authors evaluated the interaction between the two markers. On the other hand, to evaluate the systemic inflammation caused by intestinal dysfunction the authors quantified another different set of biomarkers: CRP, IL-1, IL-4, IL-10, INF-γ and TNF. To understand whether damage is more important in the elderly or whether infection is more frequent in intestinal damage after SARS-CoV, the authors performed separate analyses of elderly and young patients and also analyzed patients with and without associated bacterial co-infection separately.

## Methods

This study was approved by the Institutional Review Board of Hospital das Clinicas da Faculdade de Medicina da Universidade de São Paulo (approval number: CAAE 30,417,520.0.0000.0068). This study is not a clinical trial; it is an observational study. All patients or their legal guardians gave written informed consent as approved by IRB. The procedures followed in this study were in accordance with the principles outlined in the “Declaration of Helsinki”.

### Patients

The authors studied 87 patients (46 patients older than 61 years and 41 patients under 60 years) from the Hospital das Clínicas of the Faculty of Medicine of the University of São Paulo-Brazil with severe SARS-CoV-2 infection. Clinical evaluation revealed 32 patients with COVID-associated bacterial infection.

### Inflammatory plasma markers

Blood was collected from all patients upon admission to the hospital. Plasma levels of intestinal Fatty Acid Binding Protein (iFABP) were determined by competitive Enzyme-Linked Immunosorbent Assays (ELISA-MyBioSource; MBS 281,491) (MyBioSource, San Diego, California, USA. Plasma cytokines levels of: Interferon-gamma (IFN-γ), Tumor Necrosis Factor-alpha (TNF-α), Interleukins 10, 1 beta and 4 (IL-10, IL-1β and IL-4 respectively) were determined using MILLIPLEX Human Cytokine/Chemokine Magnetic Bead Panel (HCYTOMAG-60 K EMD Millipore Corporation; Billerica, Massachusetts [MA], USA) according to the manufacturer's instructions. Plasma levels of LBP (LPS‐binding protein), a marker of intestinal leakiness, were determined by Enzyme-Linked Immunosorbent Assays (ELISA-MyBioSource, MBS 2513,363).

Statistical analyses were performed using Graph Pad Prism version 8. The normality of the data distribution was determined by the Kolmogorov-Smirnov, Shapiro-Wilk and D´Agostino and Pearson tests and rejects the normality. Results were analyzed using the Mann-Whitney test. A p-value < 0.05 was considered significant.

## Results

The authors analyzed 87 patients who were hospitalized for SARS-COV-2 and observed that the clinical characteristics of patients separated into young and elderly patients did not show differences (Table 1). It is important to emphasize that there were no differences in CRP and leukocyte parameters, therefore without an evident inflammatory difference. However, there was a difference in mortality between the young and elderly groups, with a higher mortality rate in the elderly (Table 1). However, the authors noted in the cytokine data that there was a difference in TNF.

**Table 1:** Clinical characteristics of patients young and elderly.

**Characteristics**	**All**	**Age group**	
	**All**	**Young**	**Old**
Pacients	87	40	47
Man, n (%)	36 (40 %)	16 (40 %)	20 (42.5 %)
Age	61 ± 15	47 ± 8	72 ± 8
Leukocytes	8352 ± 2928	9010‒3502	7750‒2812
CRP	192 ± 98	180‒133	214‒75
ICU, n (%)	50 (55 %)	22 (55 %)	26 (55 %)
Mortality, n (%)	25 (28 %)	5 (12.5 %)	20 (42 %)^a^

Patients were analysed by age difference. Man represent 40 % of the patients. Inflammatory response was showed by leukocytes and c-ractive protein. Clinical evolution was despicted by ICU hospitalization and mortality.

a p < 0.05 comparing old vs. young.

Analyzing patients in relation to the presentation of an associated bacterial coinfection, there is again no difference between the various parameters reported (Table 2). However, the mortality rate is higher in patients with bacterial coinfection (Table 2).

**Table 2:** Clinical characteristics of patients with bacterial co-infection or without bacterial infection.

**Characteristics**	**All**	**Bacterial Co-Infection**	
		**No Bact infection**	**Infected**
Pacients	87	24	32
Man, n (%)	36 (40 %)	12 (50 %)	10 (31 %)
Age	61 ± 15	59 ± 17	65 ± 13
Leukocytes	8352 ± 2928	7220 ± 1500	9192 ± 2984
CRP	192 ± 98	95 ± 12	220 ± 98
ICU, n (%)	50 (55 %)	21 (88 %)	29 (91 %)
Mortality, n (%)	25 (28 %)	5 (21 %)	19 (60 %)^a^

Patients were analysed by the presence of bacterial co-infection. Man represent 40 % of the patients. Inflammatory response was showed by leukocytes and c-ractive protein. Clinical evolution was despicted by ICU hospitalization and mortality.

a p < 0.05 comparing infected vs. not infected.

**Fig. 1:**
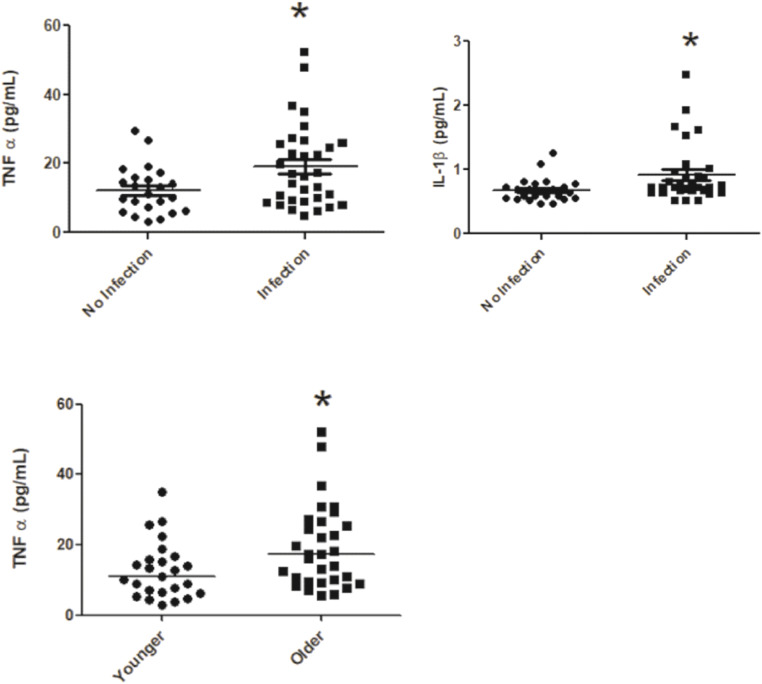
Plasma levels of TNF-α (A; *p* = 0.0398), IL-1 alfa (B; *p* = 0.0086) on groups no infection (*n* = 23) and infection (*n* = 31). Plasma levels of TNF-α (C; *p* = 0.0224) on groups younger (*n* = 25) and older (*n* = 31).

Plasma cytokines (IFN –γ, IL-10 and IL-4) were not different in infected patients when compared to non-infected patients (Fig. 1), However, TNF-α and IL-1 β were higher in infected patients (Fig. 1A and 1B respectively).

**Fig. 2:**
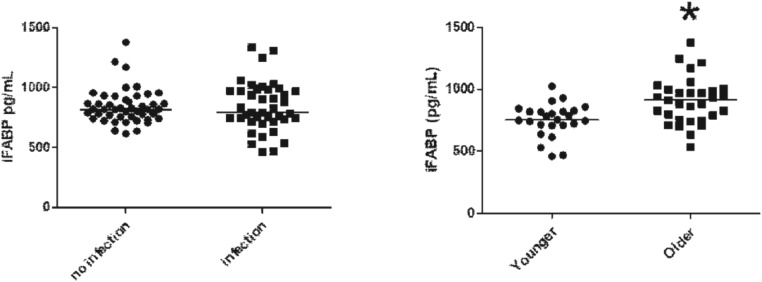
Plasma levels of iFABP on groups no infection (*n* = 46) and infection (*n* = 41) (A; *p* = 0.9830) and on groups older (*n* = 31) and younger (*n* = 25) (B; *p* = 0.0015).

TNF-α was also higher in the older patient population compared to younger patients (Fig. 1C). However, compared cytokines in an older group to a younger group, IL-1β was not different between younger and older groups (Table 3). iFABP plasma levels were not statistically different in patients with co-infection when compared to patients without co-infection (Fig. 2A); however, in the older population were found higher levels of iFABP plasma levels compared to younger patients (Fig. 2B).

**Table 3:** **Plasma levels of Cytokines.** Inflammatory response of patients during SARS-CoV infection.

**Cytokines**	**Age group**		
**(pg/mL)**	**Younger**	**Older**	p-value
IFN-γ	4.468 ± 0.871	5.650 ± 1.676	0.8432
IL-10	40.380 ± 7.413	48.490 ± 7.586	0.6326
IL-1β	0.750 ± 0.067	0.998 ± 0.166	0.0597
IL-4	8.748 ± 2.413	9.635 ± 1.762	0.1078

**Fig. 3:**
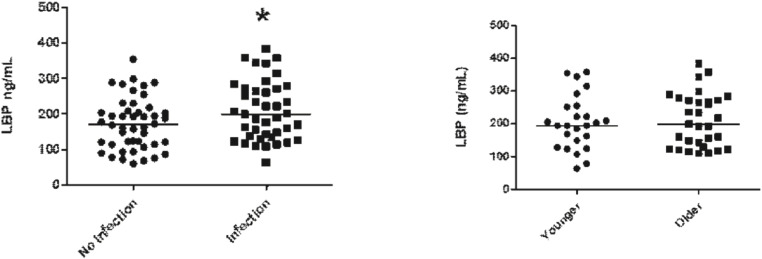
Plasma levels of LBP on groups no infection (*n* = 46) and infection (*n* = 41) (A; *p* = 0.0314) and on groups older (*n* = 31) and younger (*n* = 25) (B; *p* = 0.8756).

LBP plasma levels were higher in patients with co-infection when compared to patients without co-infection (Fig. 3A); however, when comparing LBP plasma levels in older populations with younger patients no differences could be found (Fig. 3B).

**Fig. 4:**
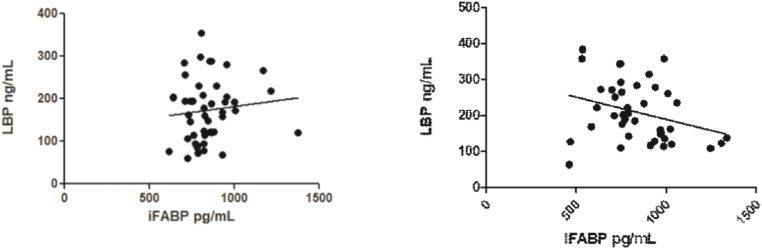
Correlation between plasma levels of LBP and iFABP (*n* = 46) on groups no infection (A; *p* = 0.4177) and infection (B; *p* = 0.0540).

The authors could not find a high correlation between the plasma levels of LBP and iFABP in patients without (Fig. 4A) or with infection (Fig. 4B).

## Discussion

In the present study, the authors observed higher plasma levels of iFABP in the plasma of older patients with SARS-CoV-2 infection when compared to younger patients. The authors also found increased plasma levels of TNF-α in the elderly compared to young patients, demonstrating a greater susceptibility to developing systemic inflammation followed by damage to intestinal epithelial cells. The literature presents data showing that patients with diarrhea and COVID-19 infection had higher TNF-α in serum.^18^ On the other hand, elderly patients have a mortality rate almost 3 times higher than young patients. Elevated inflammation and greater intestinal damage may contribute to worse outcomes in elderly patients. Increased plasma levels of ZO1 (Zonula Occludens-1), indicating damage to the intestinal tight junction complex, have been observed in patients infected with SARS-CoV-2.^19^^,^^20^

The intestinal epithelial barrier is the interface between the contents of the intestinal lumen, mainly bacteria, food, antigens, and other substances, and the bloodstream. It is a complex anatomical structure that includes an uninterrupted monolayer of epithelial cells with an intercellular junctional complex that prevents the translocation of microorganisms into the intestinal mucosa and thus into the bloodstream.^15^ Damage of intestinal mucosa has been observed in hypovolemic shock, aortic dissection, aortic surgery, Pringle maneuver (used in liver resection), hemorrhagic shock, burns, acute pancreatitis and septic shock.^16^^,^^21^, ^22^, ^23^, ^24^ In acute pancreatitis, a relationship has been found between bacteremia, infected necrosis, organ failure, and intestinal barrier dysfunction.^25^

### Patients co-infected with bacteria

Intestinal Fatty Acid Binding Protein (iFABP) can appear in the blood after intestinal damage and therefore be used as a marker of intestinal damage. .^16^^,^^17^ Bacterial translocation following intestinal damage may complicate these conditions by increasing systemic inflammation and multiple organ dysfunction.^24^ Plasma levels of LBP, a marker of intestinal leakiness, were higher in SARS-CoV-2 infected patients with systemic bacterial co-infection. Similar results have been previously reported in patients with SARS-CoV-2 infection and cardiac involvement.^26^ However, in the present study, the authors found no relationship between plasma iFABP levels and the occurrence of systemic bacterial coinfection. Therefore, the greater amount of plasma LBP in patients with bacterial co-infection cannot be explained by greater intestinal epithelial damage and the occurrence of bacterial translocation. Patients with bacterial coinfection in this study probably did not acquire the bacteria by intestinal translocation. The authors also found no correlation between the two markers of intestinal damage in SARS-CoV-2 infected and non- SARS-CoV-2 infected patients. Despite no differences in the intestinal barrier, a slight increase in inflammation and higher mortality were found in bacteria co-infected patients.

### Elderly patients

As noted, aging is associated with increased morbidity and mortality from systemic inflammation, as found in acute pancreatitis.^27^ This increased severity of inflammatory/infectious disease in the elderly has been attributed to the increased number of comorbidities in this population,^28^ however, some authors consider aging as an independent prognostic factor.^29^

This greater severity of inflammatory disease in the elderly population is not fully understood, however, some possibilities have been suggested, such as the presence of a pro-inflammatory state in the elderly population.^30^ Other authors have suggested that in the elderly population, specific changes in organs, such as the lungs, could cause excessive inflammation. Thus, prior lung injury is responsible for a second wave of cytokines released during a systemic infection, which may further worsen the systemic inflammatory process.^30^ For instance, in the elderly population with acute pancreatitis, increased intestinal damage and therefore increased bacterial translocation have been implicated in worse disease severity. Indeed, elderly patients, despite presenting local lesions similar to those observed in younger patients, present with more severe acute pancreatitis.^27^

The authors recently demonstrated that bacterial translocation in acute pancreatitis in the elderly population is related to increased pancreatic infections and severity of acute pancreatitis in a model of experimental pancreatitis model.^31^ In the inflammatory process, intestinal barrier dysfunction has also been implicated as the initial cause of multiple organ failure in this situation.^32^ On one hand, cytokine production in aging patients is similar to that of younger patients.^33^ On the other hand, the experimental work of the studied group showed higher expression of the Cox-2 gene in the intestine of older rats with acute pancreatitis compared with young rats, indicating increased intestinal inflammation.^34^

However, differences between younger and older populations related to intestinal barrier dysfunction in COVID-19 infection have not been previously investigated. In a recent review, the intestinal vulnerability of the elderly was considered to underlie the higher mortality of SARS-CoV-2 in this population.^35^ The intestinal barrier contributes to the pathophysiology of SARS-CoV-2 infection events, causing severe systemic complications. There is evidence indicating that SARS-CoV-2 disrupts the biological, mechanical, and immunological integrity of the intestinal barrier. Intestinal tissue also presents ACE2, allowing the virus to invade and destroy cells; this fact, associated with severe systemic inflammation, leads to damage to the intestinal epithelium. Enterocyte Tight Junctions (TJs) are disrupted and apoptotic death of intestinal epithelial cells is increased, leading to increased intestinal permeability.^36^

### Limitations of this study

The relatively low number of patients and the lack of information related to the resolution of intestinal integrity in older and younger patients.

## Conclusion

In conclusion, the present study suggests that intestinal barrier dysfunction in SARS-CoV-2 infections is mainly due to damage to intestinal enterocytes. In the elderly population, the authors also observed an increase in intestinal epithelial damage and increased TNF-α levels, however, with similar bacterial translocation. These changes are followed by higher mortality in elderly patients. Patients infected with bacteria had a higher degree of inflammation and mortality, although the authors did not find a mathematical correlation between the two markers of intestinal damage.

## Funding

FAPESP.

## Declaration of competing interest

The authors declare no conflicts of interest.
